# Evaluation of an ultraviolet room disinfection protocol to decrease nursing home microbial burden, infection and hospitalization rates

**DOI:** 10.1186/s12879-017-2275-2

**Published:** 2017-03-03

**Authors:** Christine R. Kovach, Yavuz Taneli, Tammy Neiman, Elaine M. Dyer, Alvin Jason A. Arzaga, Sheryl T Kelber

**Affiliations:** 1 University of Wisconsin-Milwaukee, 1921 East Hartford Avenue, Milwaukee, WI 5321 USA; 2 Department of Architecture, Uludag University, 16059 Görükle, Bursa, Turkey; 3 Jewish Home and Care Center, 1414 N. Propect Avenue, Milwaukee, WI 53202 USA

**Keywords:** Nursing home, Long-term care, Infection, Pneumonia, Prevention, Environment

## Abstract

**Background:**

The focus of nursing home infection control procedures has been on decreasing transmission between healthcare workers and residents. Less evidence is available regarding whether decontamination of high-touch environmental surfaces impacts infection rates or resident outcomes. The purpose of this study was to examine if ultraviolet disinfection is associated with changes in: 1) microbial counts and adenosine triphosphate counts on high-touch surfaces; and 2) facility wide nursing home acquired infection rates, and infection-related hospitalization.

**Methods:**

The study was conducted in one 160-bed long-term care facility. Following discharge of each resident, their room was cleaned and then disinfected using a newly acquired ultraviolet light disinfection device. Shared living spaces received weekly ultraviolet light disinfection. Thirty-six months of pretest infection and hospitalization data were compared with 12 months of posttest data. Pre and posttest cultures were taken from high-touch surfaces, and luminometer readings of adenosine triphosphate were done. Nursing home acquired infection rates were analyzed relative to hospital acquired infection rates using analysis of variance procedures. Wilcoxon signed rank tests, The Cochran’s Q, and Chi Square were also used.

**Results:**

There were statistically significant decreases in adenosine triphosphate readings on all high-touch surfaces after cleaning and disinfection. Culture results were positive for gram-positive cocci or rods on 33% (*n* = 30) of the 90 surfaces swabbed at baseline. After disinfectant cleaning, 6 of 90 samples (7.1%) tested positive for a gram-positive bacilli, and after ultraviolet disinfection 4 of the 90 samples (4.4%) were positive. There were significant decreases in nursing home acquired relative to hospital-acquired infection rates for the total infections (*p* = .004), urinary tract infection rates (*p* = .014), respiratory system infection rates (*p* = .017) and for rates of infection of the skin and soft tissues (*p* = .014). Hospitalizations for infection decreased significantly, with a notable decrease in hospitalization for pneumonia (*p* = .006).

**Conclusions:**

This study provides evidence that the pulsed-xenon ultraviolet disinfection device is superior to manual cleaning alone for decreasing microbes on environmental surfaces, as well as decreasing infection rates, and the rates of hospitalization for infection. Results suggest that placing a stronger emphasis on environmental surface disinfection in long-term care facilities may decrease nursing home acquired infections.

## Background

People residing in nursing homes are at increased risk for infection [1, 2]. Transmission occurs through transfer from colonized or infected individuals, transfer from the hands of health care workers, and contact with contaminated objects in the environment [3].

Microbial contamination of environmental surfaces in nursing homes is well documented. Plate counts have been positive in 78% of samples, Methicillin resistant staphylococcus aureus (MRSA) has been found on 16% of surfaces, norovirus on 6%, and vancomycin resistant enterococcus on 23% [4–6]. Most gram-positive and many gram-negative bacteria can persist on dry surfaces for months [7, 8]. Chemical disinfectants are not highly effective at eradicating viruses and spore producing bacteria such as Clostridium, and thoroughness of cleaning high-touch surfaces varies [9].

Little empirical evidence is available regarding whether decontamination of high-touch environmental surfaces in nursing homes substantially impacts infection rates or resident outcomes. MRSA environmental contamination in one study was associated with less frequent cleaning of shared spaces and less time spent cleaning per room, suggesting that modifying cleaning practices may reduce both MRSA environmental contamination and infection rates [6]. One group studying a norovirus outbreak attributed the short length of the outbreak to frequent cleaning of environmental surfaces and use of contact precautions [5]. Two older studies found that comprehensive environmental cleaning with a variety of disinfecting products was associated with a decrease in new rates of Clostridium difficile (C. diff) [10, 11]. Manual cleaning with approved disinfectants is the current standard of disinfection, but effectiveness is difficult to maintain because of incomplete disinfection, especially of high-touch surfaces that serve as vectors for transmission [4]. More research is needed to understand whether specific cleaning practices affect contamination, infection rates, and comorbidity. Based on this rationale, we added pulsed-xenon ultraviolet disinfection to one long-term care facility’s environmental cleaning practices. The purpose of this study was to examine if ultraviolet disinfection of environmental surfaces is associated with: 1) changes in microbial counts and adenosine triphosphate (ATP) hygiene measures on high-touch surfaces; and 2) changes in facility-wide resident nursing home acquired infection rates, and infection-related hospitalization.

## Methods

### Setting and design

The study was conducted in a 160-bed long-term care facility in the upper Midwest. One floor of 34 beds is devoted to rehabilitation and 126 beds provide skilled long-term care. The study involved a pretest-posttest design with repeated measures. Thirty-six months of pretest data were compared with 12 months of posttest data. Environmental surfaces and facility-wide rates of infection and hospitalization were the units of analysis.

### The ultraviolet disinfection protocol

The Xenex Germ-Zapping Robot™ (Xenex Disinfection Services) is a portable device that produces a high intensity flashing light, delivered in millisecond pulses, from across the entire disinfecting spectrum (from 200 to 320 nm). This germicidal UV energy passes through the cell walls of bacteria, viruses and bacterial spores. The DNA, RNA, and proteins are damaged by four mechanisms. Photohydration (pulling water molecules into the DNA), photosplitting (breaking the DNA), and photodimerization (improper fusing of DNA bases) all prevent cell replication. In addition, photo crosslinking causes irreversible cell wall damage and cell death [12].

### ATP hygiene measure

Luminometers are commonly used to measure food contamination and to monitor the effectiveness of surface cleaning and demonstrate reliable performance for measuring surface cleaning effectiveness [13]. A swab sample of high-touch surfaces placed in the luminometer (Hygiena EnSURE V.2, Scigiene Inc.) measured the quantity of light generated by a bioluminescence reaction. Results, expressed as Relative Light Units (RLU), indicate the amount of adenosine triphosphate (ATP) in the sample. The presence of ATP indicates that a surface may harbor microorganisms and support bacterial growth but measures come from anything organic in the sample [14]. The ATP swabs are not able to detect UV disinfection. The organism may be killed or rendered inactive but the ATP molecule is not removed during UV exposure. The luminometer is not meant to replace microbial testing, but can provide results in 15 s. Sampling for ATP was done before cleaning and repeated again after the room had received both cleaning and ultraviolet room disinfection.

### Cultures

To gain a better understanding of the effectiveness of the ultraviolet disinfection on true microbial contamination, culture swab samples were collected for culture from 30 consecutive discharge rooms at three time points: baseline; post cleaning, and post ultraviolet disinfection. This yielded a total of 90 cultures from 30 rooms, and three locations for each of the three different time points. Established laboratory-based procedures were used and included swabbing blood agar plates in a four quadrant fashion and placement into an O_2_ incubator for 48 h. Gram stain, morphology, and colony counts of gram-positive bacilli from 1+ to 4+ were reported.

### Rates of infection and hospitalization for infection

Each incidence of hospital acquired and nursing home acquired infection and hospitalization for an infection for each of the 48 months was obtained from records maintained by the facility. Infection rates were expressed per 1,000 resident-care days per month. Infections were considered hospital acquired if symptoms started less than 48 h after transfer from the hospital to the nursing home. Nosocomial infections were defined by the onset of symptoms 48 h or more after admission to the nursing home [15]. To get a better estimate of hospitalizations for nursing home acquired infections, we excluded residents who were readmitted to the hospital within 30 days for infection.

### Procedures

When a resident was discharged, one of two trained research staff were notified to collect baseline ATP measures from five high-touch surfaces: bed rail, call light button, bedside table surface, bathroom toilet seat, and right bathroom faucet handle. The housekeeping staff then cleaned the room for approximately 40 min and high-touch surfaces were wiped down using a stabilized sodium hypochlorite and detergent solution cleaning agent.

**Fig. 1:**
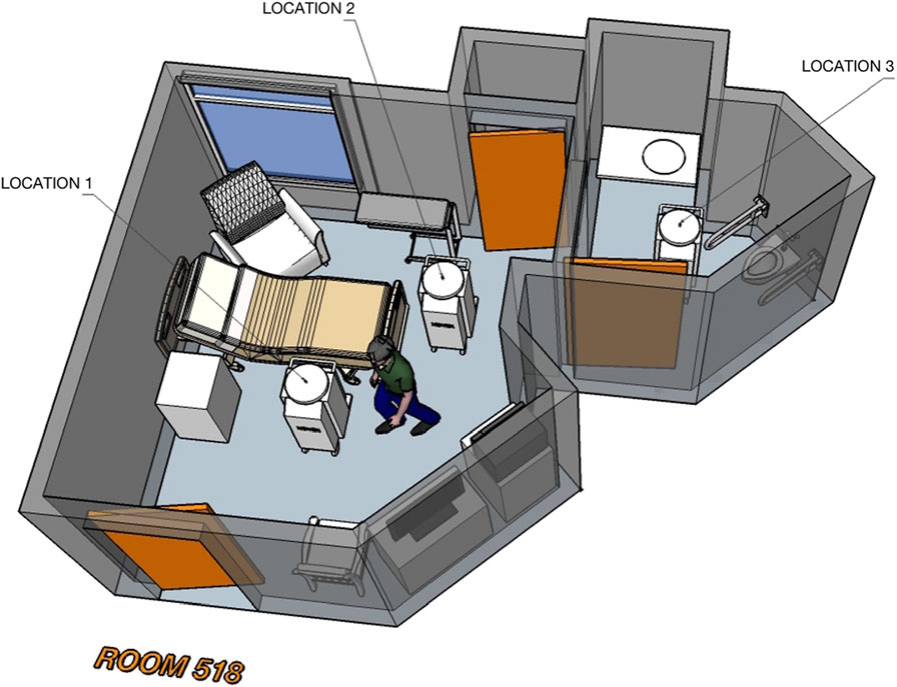
Locations for Ultraviolet Disinfection Device. Typical room layout and multiple placement of disinfection device to maximize exposure of surfaces is shown

Following cleaning and prior to ultraviolet disinfection, surfaces were prepared so that they were exposed to ultraviolet light during its operation. Call buttons, blood pressure cuffs, and telephone handsets were turned to face the device’s location. Dresser drawers, closet doors, and shower curtains were opened and blinds were closed. The ultraviolet disinfection device was preset to beam ultraviolet light for 5 min each time it was turned on and staff left the room within 15 s of the device being turned on. Figure 1 shows the three locations the device was placed for 5 min of exposure in each location. Prior to exposure in the second bedroom location some surfaces were again shifted to maximize exposure of surfaces to the pulsed light emitted by the device (e.g. handset of phone turned over). Multiple locations for the device allowed for the high intensity UV-C light to reach a different shielded surface each time, minimizing shadowed areas. For UV-C light to be effective, line-of-sight exposure is desired. However, reflected ultraviolet light has also been shown to be effective under certain circumstances in achieving decontamination of areas not exposed to direct light [16, 17]. ATP samples were again collected after the cleaning and ultraviolet disinfection were both completed.

For 30 consecutive rooms, swab samples of high-touch surfaces were collected after resident discharge at threetime points: baseline; post cleaning, and post ultraviolet disinfection. Two assistants were trained to collect culture samples of three high-touch surfaces using consistent and sterile procedures: the enabler bar, overbed table, and right bathroom faucet handle. The sterile swab was placed into a balanced isotonic solution to maintain organism viability (BD Sterile Pack Swab for surface and equipment sampling), stored in a lab refrigerator at a temperature range of 35 to 39 °F, and sent to the laboratory within 8 h. The variation in elapsed time from collection to deliver to the lab varied from 5.5 to 8 h with the first culture swabs taken at baseline having the longest elapsed time to lab delivery.

### Data analysis

ATP and culture plate data were highly skewed and analyzed with the median, range and non-parametric statistics. Differences in ATP RLU’s from baseline to post cleaning and ultraviolet disinfection were compared using the median and Wilcoxon signed rank tests. Differences in microbial cultures between baseline, post cleaning, and post ultraviolet disinfection were described using frequencies and percentages. The Cochran’s Q non-parametric test for related samples was used to compare differences in culture results between the three time points.

Our analysis of infection rates used ratios to account for the lack of independence between rates of nursing-home acquired and hospital-acquired infections. Hospital-acquired infections have become more common as medical treatments and patient complexity have increased and are the sixth leading cause of death in the United States [18, 19]. Our data showed substantial increases each year in hospital acquired infections between 2012 and 2015. Nursing homes are federally mandated to consider an infection nursing-home acquired if symptoms emerge 48 h or more after hospital discharge [15]. This siloing of infection attribution is problematic because the incubation periods for many common viral and bacterial illnesses are longer than 48 h [20, 21]. The reciprocal relationship between hospital and nursing home acquired infection has been demonstrated [22]. Using a model based on actual patient and agency data to simulate the movement of infection between hospitals and nursing homes, the influence of hospitalization on nursing home MRSA prevalence and nursing homes influence on hospital MRSA prevalence levels was demonstrated [22]. Hence, the hospital acquired infection rates were analyzed relative to nosocomial infection rates.

To analyze differences in infection rates from pre to posttest, the independent variables were the 36 months of pre disinfection time (2012, 2013, and 2014) and 12 months of post disinfection time (2015). The dependent variables are hospital acquired and nursing home acquired infection rates for the urinary tract, respiratory tract, and skin. Enteric infections occurred at such a low frequency inferential analyses could not be performed. Univariate analyses of variance (ANOVAs) were initially examined to determine if there were significant differences in rates of urinary, respiratory, and skin and enteric infections between the three pre and one post ultraviolet irradiation study periods. The effect of the ultraviolet disinfection was computed using ANOVA with an a priori contrast comparing the ratio of hospital acquired to nursing home acquired infections from pre to posttest. If the hospital acquired rate increased and the nosocomial rate decreased or stayed the same the ratio increased. If the hospital acquired rate decreased and the nosocomial rate stayed the same or decreased the ratio decreased.

Rates of infection-related hospitalization were compared from pre to posttest using Chi Square. Hospitalization rates per month were examined relative to the monthly census during that time period.

## Results

There were 247 discharges over the course of a year. All rooms received the ultraviolet disinfection protocol. In addition, shared rooms such as the dining rooms and activity rooms received daily cleaning and ultraviolet disinfection weekly. Cleaning times in the study periods remained consistent and no changes in disinfectants were made from the pre to the post study periods.

### Environmental surface changes

**Fig. 2:**
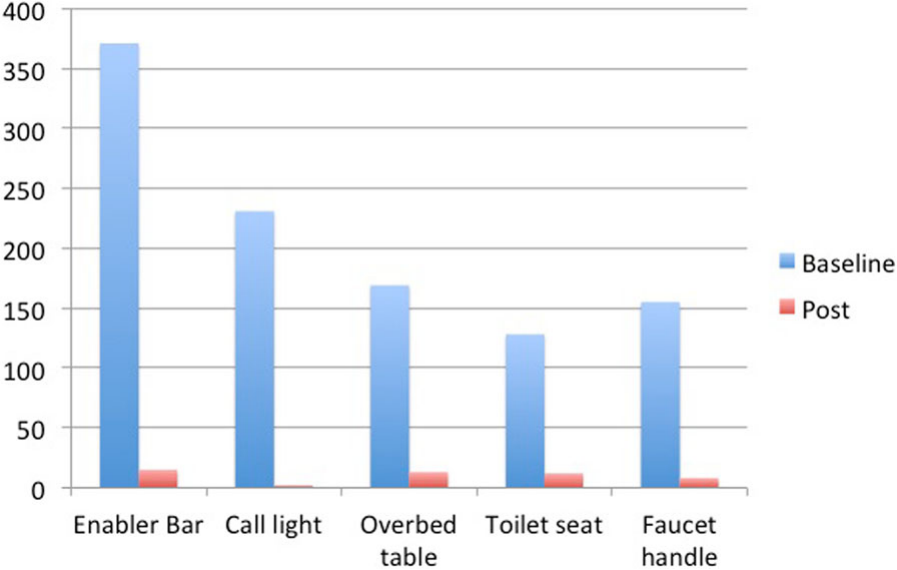
Median Differences in Adenosine Triphosphate (ATP) on High Touch Surfaces. On the horizontal axis are the surfaces sampled, and the vertical axis is expressed in Relative Light Units (RLUs)

There was a statistically significant decrease in ATP readings on all five high-touch surfaces sampled after cleaning and disinfection Table 1 and Fig. 2). Cultures taken from 30 of the rooms showed that 21 (70%) had either a gram-positive cocci or rod on one of the high-touch environmental surfaces for one testing time or more. Seventy percent of rooms (*n* = 21) tested positive for gram-positive cocci on a high-touch surface and 23% of rooms (*n* = 7) tested positive for gram-positive rods. Seventy-six percent of the enabler bars, 67% of the overbed tables, and 20% of the faucet handles tested positive at baseline.

**Table 1:** Differences in relative light units of adenosine triphosphate (atp) on high-touch surfaces

Surface	Baseline^a^	Post^b^	*p*
	Median (range)	Median (range)	
Enabler bar	371 (0–6171)	15 (0–2300)	<.001
Call light	231 (2–6054)	2 (0–1722)	<.001
Overbed table	169 (0–7396)	13 (0–1475)	<.001
Toilet Seat	128 (0–7289)	12 (0–2832)	<.001
Faucet handle	155 (1–7655)	8 (0–1411)	<.001

^a^Baseline indicates pre-cleaning measurements for ATP. ^b^Post indicates after cleaning and ultraviolet disinfection measurements for ATP

There was a statistically significantly difference between baseline, post cleaning, and post ultraviolet light disinfection time points for the enabler bar (p < .001) and overbed table (p < .001) but not for the faucet handle (*p* = .069). Culture results were positive for gram-positive cocci or rods on 33% (*n* = 30) of the 90 surfaces swabbed at baseline. After disinfectant cleaning, 6 of 90 samples (7.1%) tested positive for a gram-positive bacilli, and after ultraviolet disinfection 4 of the 90 samples (4.4%) were positive.

### Infection rate differences

**Fig. 3:**
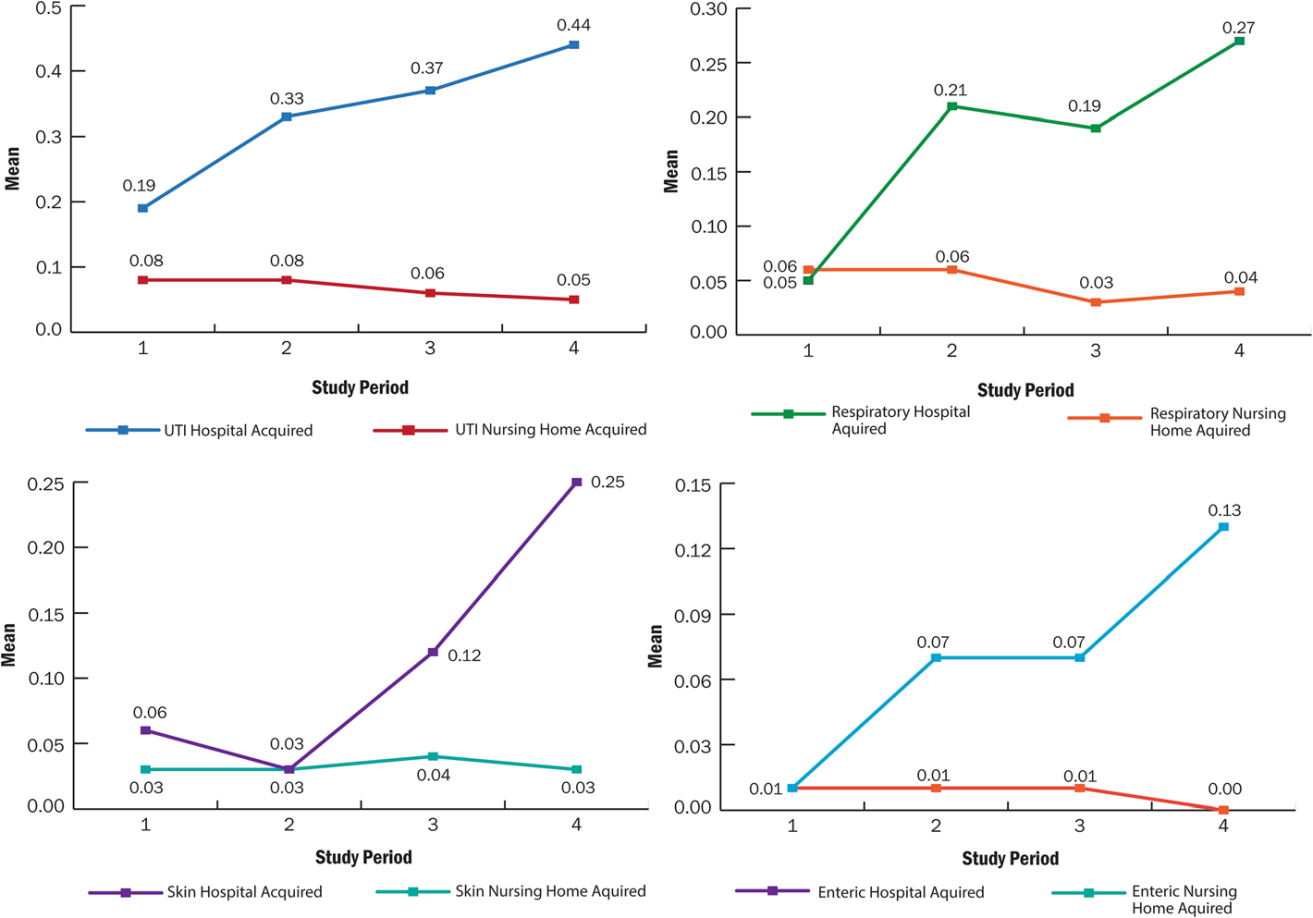
Changes in Hospital-Acquired and Nursing Home Acquired Infection Rates between Pre and Post Time Period. On the horizontal axis 1–3 are pre time periods (2012–2014), and four is the post disinfection time period (2015)

The pattern of change of hospital acquired and nursing home acquired infections is displayed in Fig. 3. Hospital acquired rates showed an increasing trend between 2012 and 2015. Comparisons of nursing home acquired rates from pretest time periods (2012–2014) to the post disinfection time period (2015) were relatively flat with small decreases from pre to posttest. The initial univariate ANOVAs, used to determine differences in rates of infections between the 3 pre and 1 post study period revealed significant interaction effects between hospital acquired and nursing home acquired rates over the four year period for respiratory system infections (F(91,44) = 4.57, *p* = .007) and skin and soft tissue infections (F(1,44) = 5.50, *p* = .003), but not for urinary tract infections (F(3,44 = 2.38, *p* = .081).

**Fig. 4:**
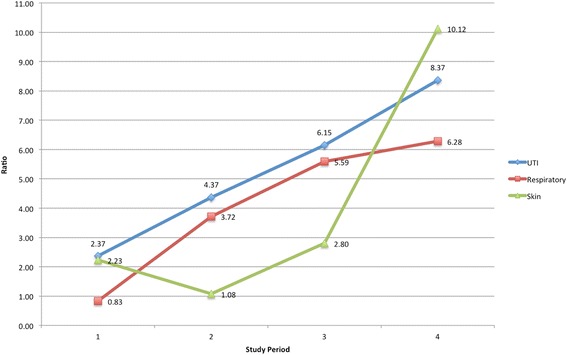
Pattern of Change Expressed as a Ratio of Hospital-Acquired to Nursing Home Acquired Infection Rates. On the horizontal axis 1–3 are pre time periods (2012–2014), and four is the post disinfection time period (2015). Increases indicate the hospital acquired rate increased while the nursing home acquired rate decreased or stayed the same

The infection ratio, computed to analyze the pattern of change, revealed a significant pre-to-post difference in the ratio of hospital acquired to nursing home acquired infections for the total infection rates (F(3,44) = 5.02 *p* = .004), urinary tract infection rates (F(3,44) = 3.97 *p* = .014), respiratory system infection rates (F(3,44) = 3.76 *p* = .017) and for rates of infection of the skin and soft tissues (F(3,44) = 3.97 *p* = .014. As seen in Fig. 4, in all cases the ratio increased from pretest to posttest indicating that as the hospital acquired rate was increasing the rate of nursing home acquired infections was decreasing or staying the same.

### Infection-related hospitalization differences

Table 2 shows the decreases in hospitalizations per year for various infection-related diagnoses. There was a statistically significant decrease in hospitalizations for infection between the pre and post time periods (Chi Square (df 3) = 12.84, *p* = .006).

**Table 2:** Hospitalizations for infection

Type	Pre (2012)	Pre (2013)	Pre (2014)	Post (2015)
	f	f	f	f
Pneumonia/Respiratory	19	14	12	5
Urinary Tract	14	3	4	2
Skin/Wound	4	3	5	0
Digestive	1	2	1	2
Sepsis	4	1	5	5
Other	1	4	5	1
TOTAL	43	27	27	15

Chi Square (df 3) = 12.84, *p* = .006

## Discussion

Infection-control programs in long-term care facilities are hampered by the lack of research to support the effectiveness of such programs or individual components of programs. This study provides evidence regarding several key aspects of one infection control program. The high percentage of high-touch environmental surfaces testing positive for gram-positive bacteria is consistent with other studies that show these bacteria can live on dry surfaces for months [7, 8]. Culture results showed that the majority of high-touch environmental surfaces were effectively disinfected with standard cleaning practices. The ultraviolet disinfection decreased the number of positive cultures by an additional 2.7%. In addition, the ultraviolet light was able to reach and treat many hard and soft surfaces that are not routinely cleaned with a disinfectant. A hospital-based study showed a 22% difference between manual cleaning with a disinfectant and the ultraviolet light disinfection [4]. Another hospital-based study showed a statistically significant reduction in microbial load and elimination of vancomycin-resistant enterococci from high-touch surfaces in isolation rooms [23]. The high number of surfaces adequately disinfected by manual cleaning in this study reflects a high level of thoroughness by the housecleaning staff.

In this study the luminometer readings of ATP counts were not used to direct a second cleaning of the high-touch environmental surfaces that maintained high ATP readings after cleaning. Monitoring environmental contamination is recommended to provide timely evaluative data that can be used to direct and evaluate cleaning and decontamination activities [24–26]. Given the costs involved in both frequent ATP readings and the treatment of each occurrence of infection, an examination of the costs versus benefits of using ATP pre-post cleaning readings to remediate ineffective or incomplete cleaning is warranted.

This is the first known study conducted in a nursing home to examine the effectiveness of the pulsed-xenon ultraviolet irradiation device in decreasing microbial burden and infection rates. The changes in the ratios of nursing home acquired infection relative to hospital-acquired infections from pre to posttest provide support for the effectiveness of the pulsed-xenon ultraviolet irradiation device and are consistent with findings from other health care settings. One long-term acute care facility had a 57% (*p* = .02) drop in C difficile infection when the pulsed-xenon ultraviolet irradiation device was added to the efforts of a multidisciplinary C difficile prevention team [27]. A community-based hospital in Florida found significant decreases in facility-wide and ICU infection rates [28]. Quality improvement initiatives combined with pulsed xenon ultraviolet room disinfection reduced surgical site infections in patients undergoing total joint procedures at another community hospital from seven infections from 544 procedures to 0 from 585 procedures (*p* = .01) with an estimated cost savings of $290,990 [29].

We also found that hospitalization rates decreased significantly from the pre to posttest time periods. The decrease in hospitalizations for pneumonia was particularly substantial and important considering costs of hospitalization as well as findings that residents transferred to acute care facilities with pneumonia are at increased risk for mortality and hospital-related complications including adverse drug reactions, delirium, falls, and pressure ulcers [30–32].

It is worth noting that UV-C light exposure contributes to degradation and more rapid aging of plastics, and other nonmetal objects [33]. If ultraviolet light disinfection is to be employed on a regular basis, materials/furniture in such areas should be chosen based on ability to resist degradation from ultraviolet irradiation. While UV protective paints and coatings are another option for certain materials, application could be problematic and impractical.

Limitations of the study include the lack of a control group, the use of only one nursing home and the lack of control over some potentially relevant variables. Fluctuations from year to year in regional viral and bacterial infection rates were not controlled in this study. Antibiotic use was not controlled and new federal guidelines for antibiotic stewardship may have impacted recent antibiotic use [26]. The decision to transfer a resident with an infection to the hospital is impacted by multiple factors that were not controlled including RN turnover, environmental quality, prioritizing staff satisfaction, resident privacy, and facility visitation [34]. ATP swab results were not able to isolate the benefit of UV disinfection from standard cleaning. The possible impact of variation in elapsed time of swab culture collection to lab delivery between the three data collection time points is not known. Culture samples collected were limited to high-touch surfaces that housekeeping staff are specifically trained to thoroughly clean with disinfectant. A comparison of line-of-sight and reflected ultraviolet light’s effectiveness was not part of this study and more specific understanding of the role of reflected light in decontamination is needed.

## Conclusion

In conclusion, this study provides evidence that the pulsed-xenon ultraviolet disinfection device is superior to manual cleaning alone for decreasing microbes on environmental surfaces, infection rates, and the rates of hospitalization for infection. Additional research is needed using more sites and a true experimental design. Including cost analysis in future studies of the effectiveness of the device on clinical outcomes is recommended.

## Abbreviations

ANOVA: Analyses of variance
ATP: Adenosine triphosphate
C. diff: Clostridium difficile
DNA: Deoxyribonucleic acid
MRSA: Methicillin resistant staphylococcus aureus
RLU: Relative Light Units
RNA: Ribonucleic acid
